# ReCombine: A Suite of Programs for Detection and Analysis of Meiotic Recombination in Whole-Genome Datasets

**DOI:** 10.1371/journal.pone.0025509

**Published:** 2011-10-25

**Authors:** Carol M. Anderson, Stacy Y. Chen, Michelle T. Dimon, Ashwini Oke, Joseph L. DeRisi, Jennifer C. Fung

**Affiliations:** 1 Department of Obstetrics, Gynecology, and Reproductive Sciences and Center for Reproductive Sciences, University of California San Francisco, San Francisco, California, United States of America; 2 Department of Biochemistry and Biophysics, University of California San Francisco, San Francisco, California, United States of America; 3 Biological and Medical Informatics Program, University of California San Francisco, San Francisco, California, United States of America; 4 Howard Hughes Medical Institute, Bethesda, Maryland, United States of America; National Cancer Institute, United States of America

## Abstract

In meiosis, the exchange of DNA between chromosomes by homologous recombination is a critical step that ensures proper chromosome segregation and increases genetic diversity. Products of recombination include reciprocal exchanges, known as crossovers, and non-reciprocal gene conversions or non-crossovers. The mechanisms underlying meiotic recombination remain elusive, largely because of the difficulty of analyzing large numbers of recombination events by traditional genetic methods. These traditional methods are increasingly being superseded by high-throughput techniques capable of surveying meiotic recombination on a genome-wide basis. Next-generation sequencing or microarray hybridization is used to genotype thousands of polymorphic markers in the progeny of hybrid yeast strains. New computational tools are needed to perform this genotyping and to find and analyze recombination events. We have developed a suite of programs, ReCombine, for using short sequence reads from next-generation sequencing experiments to genotype yeast meiotic progeny. Upon genotyping, the program CrossOver, a component of ReCombine, then detects recombination products and classifies them into categories based on the features found at each location and their distribution among the various chromatids. CrossOver is also capable of analyzing segregation data from microarray experiments or other sources. This package of programs is designed to allow even researchers without computational expertise to use high-throughput, whole-genome methods to study the molecular mechanisms of meiotic recombination.

## Introduction

In sexually reproducing organisms, meiosis is the specialized type of cell division that produces haploid gametes (eggs and sperm, in humans) from diploid cells. During the first meiotic division, pairs of homologous chromosomes become physically linked, and DNA is exchanged between chromosomes by homologous recombination. This exchange of DNA can either be reciprocal, leading to a crossover (CO), or non-reciprocal, giving rise to a non-crossover (NCO) or gene conversion (GC). Proper recombination between homologs is critical for two reasons: first, the physical link between homologs helps establish their alignment on the meiotic spindle and correct segregation at the first meiotic division; and second, the exchange of DNA provides a nearly limitless source of genetic diversity [1]. Errors in recombination can give rise to aneuploid gametes (containing too many or too few chromosomes), or to deleterious chromosomal rearrangements. Such errors are common causes of infertility and birth defects in humans [2].

**Figure 1:**
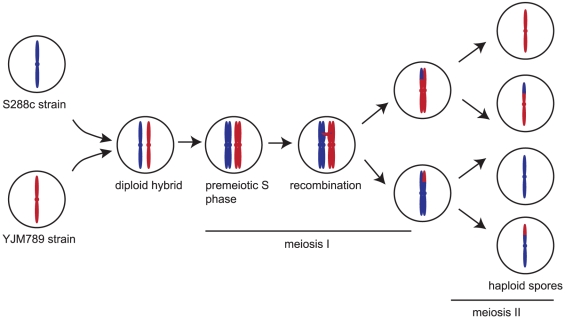
Experimental setup. Two haploid yeast strains are mated to produce a diploid hybrid. The diploid is induced to undergo meiosis, producing four haploid progeny, which are isolated for further study. For simplicity, only one chromosome per cell is shown. DNA is isolated from the spores and subjected to sequencing or microarray analysis to determine which part of each spore’s genome was inherited from each parent strain.

Much of what we know about the details of meiotic recombination comes from studies of the budding yeast *Saccharomyces cerevisiae.* In yeast, the process of sporulation produces four haploid spores from a single diploid parent cell (Figure 1). These four spores remain together as a tetrad, and can be physically separated using a micromanipulator for further study. Most studies of yeast meiotic recombination have relied on dissection of hundreds of tetrads to analyze the segregation of a small number of loci bearing nutritional or antibiotic resistance markers [3]. The large amount of hands-on time required for each experiment places a severe limitation on the number of experiments a single researcher can carry out. Many important questions about meiotic recombination, such as how cells regulate the exact location and distribution of COs, remain unanswered. Recently, our laboratory and others have developed whole-genome approaches to accelerate the study of meiosis in yeast [4], [5], [6], [7]. Using microarrays or high-throughput sequencing, we are able to detect recombination events occurring genome-wide in a single tetrad. This approach allows us to draw conclusions based on only a few tetrads rather than hundreds. In addition, we can survey the full spectrum of events occurring throughout the genome rather than limiting ourselves to a small number of marked intervals.

For whole-genome studies, we and others [4], [5], [6] mate two divergent yeast strains whose sequences differ at thousands of sites genome-wide. After sporulation and tetrad dissection, we isolate DNA from each of the four progeny and use microarray hybridization [4], [5], [7] or high-throughput sequencing [6] to genotype single-nucleotide polymorphisms (SNPs) and insertions/deletions (indels), thus determining the regions of the genome derived from each parent. Based on this information, we determine the sites of COs, NCOs, and GCs. This approach allows evaluation of multiple aspects of recombination control simultaneously and rapidly. By monitoring changes in the frequency and distribution of various types of events in mutant strains, we can characterize the roles of candidate genes and begin to understand their molecular mechanisms. For example, using microarrays we previously showed that Zip1, a synaptonemal complex protein, has a role in suppression of COs near centromeres [4]. It is important to note that these experiments only reveal recombination events between homologous chromosomes, and not events between sister chromatids that do not give rise to detectable products due to lack of sequence differences.

To obtain the best resolution for our experiments, we are now using next-generation sequencing with the Illumina/Solexa platform to genotype greater than 67,000 SNPs and indels. The median distance between markers in these experiments is 56 bp. In preparation for sequencing, a library of genomic DNA fragments derived from each spore is immobilized in a flow cell and amplified to produce clusters of approximately 1000 identical copies of each template. Hundreds of millions of clusters are then simultaneously sequenced by the addition of reversibly terminated fluorescent nucleotides, with each nucleotide bearing a distinct fluorophore. Images collected after each round of synthesis are analyzed to determine the sequence of each template. Our experiments used read lengths from 36–43 base pairs with tens of millions of reads per flow cell lane, yielding up to 27-fold average coverage of the entire yeast genome. With recent advances in read length and reads per lane, even deeper coverage can easily be obtained. As a cost-saving measure, we have also successfully used three-nucleotide “barcodes” to allow sequencing of multiple samples in a single lane, resulting in a lower, but still sufficient, 6-fold average coverage level. The high resolution of these data allows much more detailed analysis of individual recombination products than was previously possible. In addition to simple COs, NCOs, and GC tracts, we detect many complex recombination events, such as discontinuous GC tracts associated with a CO, and regions where multiple NCOs or COs cluster closely together. By carefully classifying these recombination products and measuring changes in their frequency and distribution in meiotic mutants, we hope to identify signatures characteristic of different recombination pathways. Identifying such signatures would be an important step towards understanding the mechanisms underlying CO and GC formation. For example, the Mms4-Mus81 nuclease complex is known to control formation of a subset of COs [8]. Deletion of *MMS4* was shown by high-density tiling microarray to lead to regions of frequent genotype change occurring near COs [5]. Although the reason for these changes is still unknown, the ability to detect them provides an entry point into elucidating the mechanism of CO formation by the Mms4-Mus81 pathway.

The analysis of recombination on a genome-wide scale presents two major bioinformatics challenges. The first is determining the genotype at each SNP or indel position. The second is identifying products of recombination and distinguishing between multiple recombination resolution signatures.

Regarding the first challenge, well-established methods exist for genotyping SNPs and indels by microarray; these include the programs Allelescan and ssGenotyping, which can genotype and reconstruct the segregation profile of a yeast tetrad [7], [9]. However, no similar package is available for analysis of yeast meiosis by sequencing. Next-generation sequencing generates millions of short reads that must be aligned to each of the parent genomes. Many programs exist for alignment of short reads, including Bowtie, SOAP, and Maq [10], [11], [12]. The genotype at each SNP or indel must be determined from these aligned reads. In virtually all published genotyping methods, SNPs or indels are detected de novo by identifying locations where read sequences differ from a single known reference genome [13]. Since variants can also arise due to sequencing errors or misalignment of reads, a filter or quality score threshold is usually imposed to reduce the number of false positives [12], [14]. In our yeast experiments, since the genomes of both parent strains have been sequenced, we reasoned that we could improve the accuracy of genotyping by comparing sequence reads against both reference genomes, rather than just one. To our knowledge, the only published program that compares sequence reads to two known genome sequences is a method used to genotype rice subspecies [15]. Because the rice genome is approximately 30 times larger than the yeast genome, sequencing coverage levels are generally much lower, and genotyping is also complicated by the fact that rice is diploid, and thus may have more than one allele at each locus. Due to these limitations, a sliding window encompassing 15 SNPs is used to determine final genotype calls along each rice chromosome. Use of a sliding window precludes obtaining a high-resolution picture of any single recombination event. In yeast experiments, we are able to achieve high enough coverage levels to score individual SNPs and indels without the need to resort to a sliding window. Therefore, the method used for rice is not well suited for yeast or other organisms with relatively small genomes, necessitating the development of a new method.

The second computational challenge is detecting recombination events based on the genotypes of the four progeny of a single meiosis. Manual annotation of all events is impractical given the large number of markers genotyped in each tetrad. Therefore, what is needed is a method of automating the process of finding recombination products, classifying them into different categories, and recording their location and size. Furthermore, information about the distribution of recombination events can be used to evaluate several important aspects of CO regulation. In yeast and many other organisms, COs are distributed non-randomly throughout the genome. This phenomenon, known as CO interference, ensures that COs are not clustered too closely together and that each chromosome pair sustains at least one CO. The strength of CO interference can be evaluated by measuring inter-CO distances and fitting them to a gamma distribution function characterized by a shape (γ) and scale (β) parameter [16], [17]. The γ and β parameters can be calculated using ∼250 inter-CO distances, a number detected in three wild-type tetrads by microarray or high-throughput sequencing. Another aspect of global CO control is CO homeostasis, which refers to the observation that the number of COs per meiosis tends to stay within a narrow range. When the number of initiating double-stranded DNA breaks is reduced, high CO levels are maintained at the expense of NCOs [18]. The coefficient of variation between the number of COs and NCOs per tetrad, which can be calculated based on microarray or next-generation sequencing data, provides a measurement of CO homeostasis. A third important aspect of CO regulation is the nonuniform distribution of COs along chromosomes. In particular, CO formation is repressed at centromere- and telomere-proximal regions. Regional CO suppression in these regions can typically be evaluated using whole-genome data from one to three tetrads. Computational tools are needed to perform all of these analyses of recombination control. The ssGenotyping package developed for analysis of yeast tetrads by microarray includes tools to perform some of the functions described above [9]. ssGenotyping identifies recombination events, but it does not classify them in detail. ssGenotyping is also capable of analyzing certain aspects of CO distribution, such as interference.

Here we introduce ReCombine, a package of programs developed in our lab to analyze meiotic recombination on a whole-genome level using either microarray or high-throughput sequencing data. For sequencing experiments, ReadAligner and GenotypeCaller are used to align short sequence reads to the two parent genomes and to determine the genotype of SNPs and indels. These programs can accept short sequence reads from a variety of sequencing platforms. The resulting segregation profile is then analyzed using the program CrossOver, which can also accept segregation data from microarray experiments as input. CrossOver detects various types of recombination events including COs, NCOs, and GCs, classifies them into categories, and reports many parameters including their frequency, distribution and conversion tract length. We previously used an earlier version of CrossOver to detect and analyze the major types of recombination events in microarray data [4]. The redesigned version of CrossOver presented here is capable of much more sophisticated sorting of recombination products than the previous version. Results of CrossOver also include assessments of several aspects of CO regulation, including measurements of CO interference, CO homeostasis, and regional repression of COs near centromeres and telomeres.

We demonstrate here the use of all three programs to analyze two wild-type tetrads sequenced in our laboratory, and one wild-type tetrad sequenced by Qi and co-workers [6] using a different hybrid strain and different sequencing technology. We also show the use of CrossOver to analyze recombination events in a large published microarray data set. These programs constitute a complete toolkit for using raw sequence reads to analyze recombination, which will allow even labs without bioinformatics expertise to carry out genome-wide studies of meiosis in yeast.

## Results

### Approach

Our overall strategy for data analysis consists of two major steps. First, we use the newly developed programs ReadAligner and GenotypeCaller to align sequence reads to the parent genomes and determine genotypes of SNPs and indels for each spore of a tetrad. Second, we use a redesigned CrossOver program to detect and analyze recombination events from all four spores of the tetrad. As we show below, the latter step can also be carried out with microarray data.

### Read alignment

**Figure 2:**
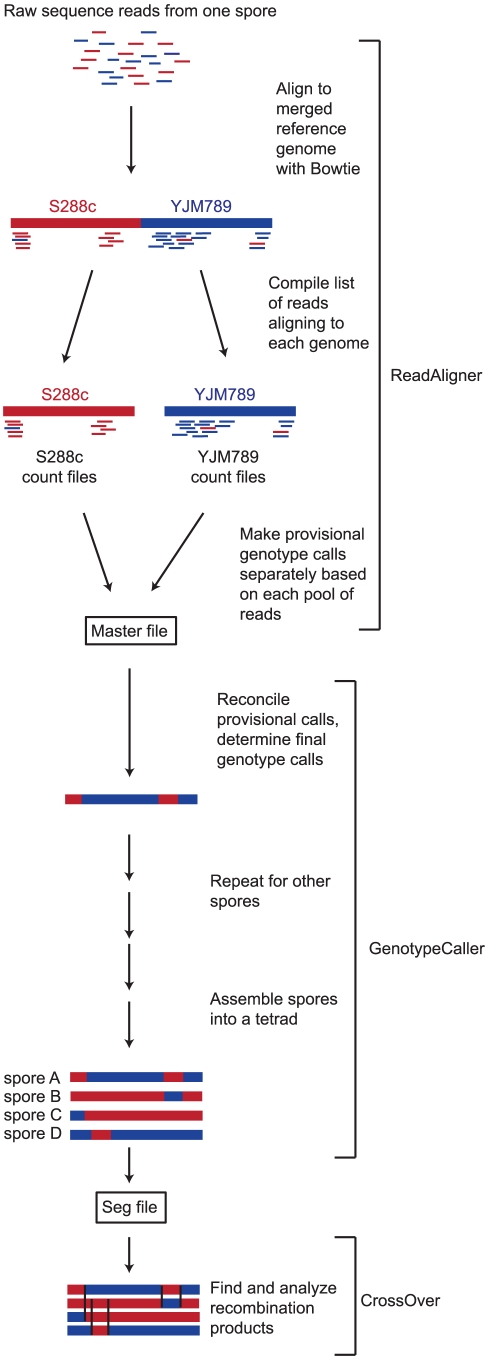
Data analysis pipeline. The figure shows the procedure as performed for a S288c/YJM789 hybrid, but other hybrid strains can also be used. Short sequence reads from one spore are first aligned against a merged reference genome containing both S288c and YJM789 sequences. For each read, the position of the best alignment is noted. Reads with more than two valid alignments within the merged reference genome are discarded. The read alignments are then divided into two separate pools: those that aligned within the S288c genome and those that aligned within the YJM789 genome. Reads aligning to the S288c genome do not necessarily match the S288c sequence; this is true because the “best” alignment reported by Bowtie is not guaranteed to be the best possible alignment when multiple mismatches are present, particularly if they fall in the low-quality end of the read. Each pool of reads is recorded in a “Count file” for downstream analysis. The information in the Count files is used to make provisional genotype calls at each SNP or indel position, taking into account the number of reads aligning to that position in a given reference genome as well as their sequences and base quality scores. These provisional genotype calls are recorded in “Master files.” The two provisional calls recorded in the Master file are then reconciled to determine the final genotype call at each SNP or indel. After this process is carried out for each spore in a tetrad, information from all four spores is cross-referenced and any SNPs or indels not genotyped in all four spores are discarded. A “Seg file” is produced listing the genotype of all four spores at each SNP or indel. Finally, the Seg file is analyzed to determine the locations of COs and GCs, GC tract lengths, and various other features of meiotic recombination.

In the first step of our analysis, sequence reads are mapped to the two parental genomes using the program ReadAligner (Figure 2). Our laboratory uses the S96 strain, which is a close relative of the common laboratory strain S288c, and YJM789, a strain originally isolated from the lung of an AIDS patient [19]. Both strains have been fully sequenced by traditional methods, and their sequences differ by 0.6%, with the differences consisting of ∼60,000 single-nucleotide SNPs and ∼6,000 indels [20]. Throughout this manuscript, we describe the procedures as performed for S288c x YJM789 hybrid progeny; however, ReCombine is also capable of analyzing data from other hybrid progeny, as long as one of the two parents is S288c. We also describe below the analysis by ReCombine of previously published data from an RM11-1a x S288c tetrad.

ReadAligner uses the Bowtie short read aligner [10] to align raw sequence reads to a merged reference genome consisting of both S288c and YJM789 sequences. A merged reference genome is used to allow reads containing sequence polymorphisms to align to the best-matched parental genome. Bowtie parameters are set such that the single best alignment is reported for each read. Reads containing no sequence polymorphisms will align equally well to both single genomes in the merged reference genome, resulting in two valid alignments; Bowtie randomly selects one to report. Our Bowtie settings instruct the program to discard any reads with more than two valid alignments to the merged genome, since they cannot be unambiguously assigned to a specific location. Thus reads aligning to exact repeats are excluded (e.g. telomere repeats, rRNA sequences). Bowtie alignment results for the merged reference genome are then separated into two pools corresponding to the parent genomes, S288c and YJM789, for downstream SNP and indel analysis. The results are contained in “Count files” (Tables S1 and S2) listing the number of reads covering each SNP or indel position. Note that it is possible, though not common, for a read to align to one genome but match the sequence of the other; this usually occurs because Bowtie does not guarantee that the reported alignment is the best possible one if all valid alignments contain mismatches, particularly if they occur in the right (low-quality) end of the read. Therefore, as well as tabulating the number of reads covering a given SNP or indel position, the count file also records whether the sequence of each read at each SNP or indel position matched the S288c or YJM789 reference genome.

ReadAligner also carries out a separate alignment for the sole purpose of genotyping telomere-adjacent SNPs. This is necessary because many of the telomere-proximal regions are missing from the published sequence of YJM789, and hence can only be evaluated by alignment to the S288c genome. ReadAligner uses a separate list of SNPs in these regions compiled from our resequencing of the YJM789 strain.

### Genotyping SNPs and Indels

In the second step of our analysis, initial SNP and indel genotypes are provisionally assigned based on the Count files. This is performed separately for reads aligning to each reference genome, and the two provisional calls are reconciled at a later step. At most locations, the reads aligning to a SNP or indel match only one of the two parental genotypes, and assigning a genotype is straightforward. However, we also find many cases in which a mixture of reads matching both genotypes (or neither genotype) align to certain SNP or indel positions. In our highest-coverage sequencing reactions, this occurs at ∼3% of SNPs and ∼17% of indels. One reason this commonly occurs is sequencing error; since mismatches are tolerated in the alignment process, a read can still align to the correct location even if it has a few wrong bases. Even if sequencing were completely error-free, some ambiguous situations would still be expected to occur due to the existence of repetitive or partially homologous regions within a genome. For example, a read derived from a Ty1 element on chromosome 1 in the S96 genome may also align mistakenly to a Ty1 element on a different chromosome in the YJM789 genome. Since the read is actually derived from chromosome 1 in this example, it would confound the proper genotyping of the other chromosome. Adding to this problem is the fact that the YJM789 genome sequence is not complete, and regions containing repetitive sequences are especially likely to be missing from the published sequence. Thus, when we attempt to align reads derived from these missing regions to a merged reference genome, they are very likely to align to the S288c genome even if they are derived from YJM789.

In order to resolve these ambiguous situations, we take advantage of quality scores produced by the Solexa/Illumina Pipeline software [21]. The software assigns a quality score to each base of every read, which represents an estimate of the likelihood of a wrong base call in the sequencing reaction at that position. We use these base quality scores to calculate a cumulative quality score for each of the expected genotypes at every SNP or indel position (Figure S1). A user-defined quality score threshold is applied to control the stringency of genotype calling. For each SNP marker, if the base with the highest cumulative quality score is above the quality score threshold, then the SNP is genotyped as that base based on reads aligning to that single reference genome. An analogous process is carried out for indel markers (details are given in Materials and Methods). The genotype is provisionally called as S96, YJM789, or neither. We refer to the genotype calls at this stage as “provisional” because they are based on reads aligning to only one reference genome; at a later step, the two provisional calls for each position are reconciled, yielding a single final call. During this initial genotyping process, if none of the nucleotide scores of a SNP or indel exceeds the threshold, the marker is not genotyped at that position based on reads aligning to that reference genome, resulting in a call of “neither.” Provisional genotype calls based on reads aligning to both reference genomes are placed in a single “Master file” (Tables S3 and S4). Next, the program GenotypeCaller reconciles the two separate provisional genotype calls from the reads aligning to each reference genome, producing a single final genotype call for each marker position (rules for reconciliation are given in Materials and Methods).

In order to map recombination events among four spores of a single tetrad, only markers that are genotyped in all four spores are used, as it is impossible to unambiguously determine a gene conversion event at a specific marker location if only a subset of the four spores are genotyped at that site. Therefore, any SNPs or indels not genotyped in all four spores are discarded at this point. GenotypeCaller produces a “Seg file,” containing a list of markers genotyped in all four spores, along with the genotype of each spore at each position (Table S5). The Seg file is used for analysis of recombination by CrossOver. The ReCombine package also includes plotting tools that use the Seg file to produce a graphical representation of the segregation of an entire tetrad or of any desired region of the tetrad.

### Detection of Recombination Events by CrossOver

**Figure 3:**
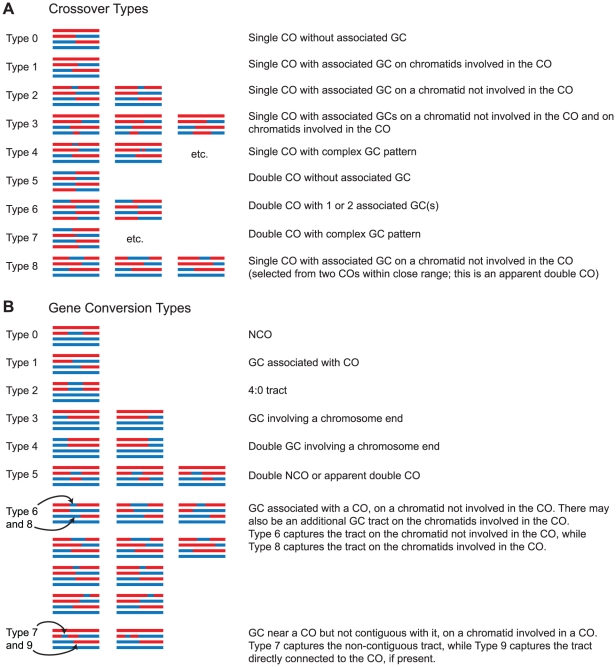
CrossOver output categories. Examples are shown of each type of recombination event that can be identified by the CrossOver program. Note that a single event may contain both a CO and a GC; for example, a Type 1 CO always contains a Type 1 GC tract, and a Type 2 CO always contains a Type 6 GC.

**Figure 4:**
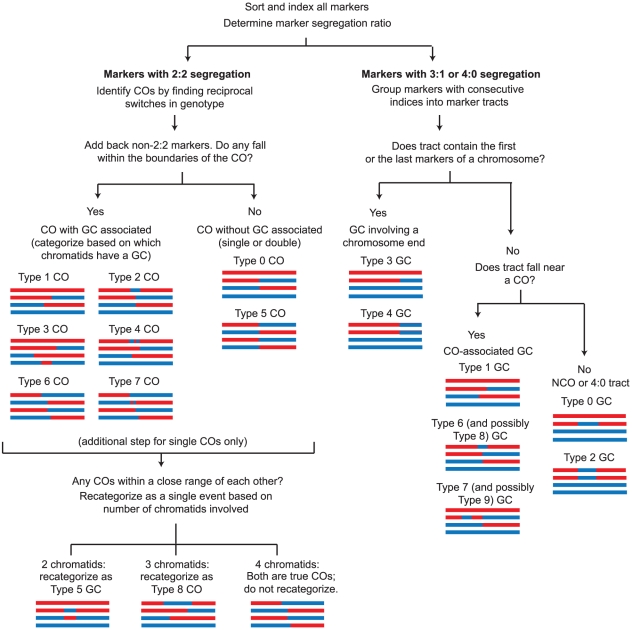
Overview of CrossOver logic. This figure shows the overall strategy used to find and classify recombination events. Additional details are given in Figure S3. Initially, all markers in a tetrad are categorized by whether they show 2∶2 segregation. The non-2∶2 markers are set aside, and 2∶2 markers are searched for locations where markers undergo a reciprocal genotype change. These are locations of COs. The program then determines whether any non-2∶2 markers fall within the boundaries of the COs; if so, these are considered GCs associated with a CO, and are categorized based on which chromatids are involved. For single COs only, the program finds any pairs of COs located within a user-defined distance of each other, and re-classifies them as single events rather than as two COs. Next, non-2∶2 markers not associated with COs are considered. Adjacent markers are grouped together into conversion tracts. If a conversion tract falls within a user-defined distance of a CO, it is categorized as a CO-associated GC. If not, it is categorized as an NCO or a 4∶0 tract.

**Figure 5:**
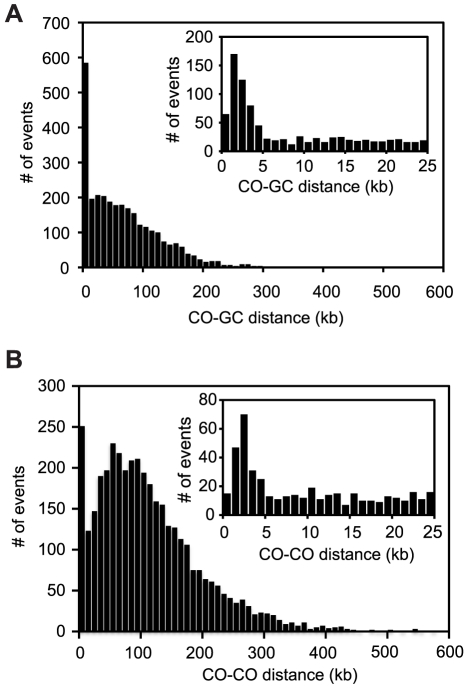
Inter-event distances. (**A**) Distances from each CO to the nearest GC tract on each side are shown for 48 wild-type tetrads, which includes tetrads *wt*x29 and *wt*x30, sequenced in our lab, and 46 wild-type tetrads genotyped by Mancera et al. by microarray [5]. The inset shows a close-up view of events falling within 25 kb of each other. A distinct subset of CO-GC distances falls below 5 kb. (**B**) Distances between pairs of adjacent COs are shown for the same set of tetrads analyzed in (A). The inset shows a close-up view of events falling within 25 kb of each other. A distinct subset of inter-CO distances falls below 5 kb.

In the third step of our analysis, the CrossOver program is used to analyze recombination events. CrossOver scans through a Seg file to identify nine categories of COs and ten categories of GCs (Figure 3). A brief summary of the logic employed by the program is shown in Figure 4 and described here; additional details are given in Materials and Methods. The program initially identifies COs as locations where adjacent markers undergo a reciprocal genotype switch (Figure 4). GC tracts, which are regions of non-2∶2 segregation, are then identified. See Materials and Methods for a detailed description of how the location of each type of event is reported. Conversion tracts that overlap with a CO are considered “CO-associated” GC tracts, and are assumed to have arisen from heteroduplex DNA created at a double Holliday junction during formation of the CO [22]. However, when a GC tract occurs near a CO but is not connected to it, the interpretation is less straightforward. Such an event could result from repair of heteroduplex DNA during resolution of a CO (and thus be part of a single event, a Type 7 GC), but it also might represent an independent NCO (a Type 0 GC). In wild-type cells, we find that the CO-to-GC distances fall into two distinct populations: the median CO-to-GC distance is 56 kb, but a distinct cluster of distances occurs below 5 kb (Figure 5a). We hypothesize that conversion tracts appearing within 5 kb of a CO arise from the same double-strand break (DSB) that creates the CO. Therefore, when classifying GC tracts near a CO, CrossOver applies a user-defined range, set to 5 kb by default. GC tracts occurring within 5 kb of a CO are considered CO-associated, while GC tracts outside that range are considered independent events. The interpretation of closely spaced COs is similarly ambiguous. In many cases, two COs that occur closely together could be interpreted alternatively as a single CO with an associated GC on a different chromatid (a Type 8 CO), or as a double NCO (a Type 5 GC) (Figure 3), rather than as two individual COs. Our analysis of inter-CO distances shows that a distinct subset falls under 5 kb (Figure 5b). Assuming closely spaced COs to be rare under wild-type levels of CO interference, we hypothesize that the small population of apparent double COs with an inter-CO distance under 5 kb are actually not double COs, but recombination events arising from a single DSB. We further analyzed these events by determining the distribution of CO pairs involving 2, 3, or 4 chromatids. For COs in wild-type cells, this ratio has previously been shown to be 1∶2∶1 [4], [23]. This reflects the fact that there is no chromatid interference; that is, a CO between two nonsister chromatids does not influence the probability of those chromatids being involved in an adjacent CO. We found that the ratio of 2-, 3-, and 4-strand double COs among pairs of apparent COs less than 5 kb apart was 1.0∶0.7∶0.3, a significant deviation from the expected ratio (Table 1). In contrast, CO pairs in which the COs are at least 5 kb apart show a ratio of 1.0∶1.9∶ 0.9, which does not deviate significantly from the expected ratio of 1∶2∶1. These results are consistent with the model that events occurring within 5 kb of each other are not true double COs. Therefore, as for CO-associated GC tracts, CrossOver applies an inter-CO distance, set to 5 kb by default, to decide whether an apparent double CO is actually the product of a single recombination event. If three or more COs are located in close proximity, the program alerts the user to manually inspect the region. Such events are rare in wild type tetrads (five events in the 46 wild type tetrads described below).

**Table 1:** Analysis of closely spaced COs.

	Number of chromatids involved in the two COs				
CO pairs considered	2 chromatids	3 chromatids	4 chromatids	ratio	p-value
All CO pairs	1043	1883	926	1.0∶1.8∶0.9	0.01
COs within 5 kb of each other	94	67	32	1.0∶0.7∶0.3	4×10^−13^
COs at least 5 kb apart	949	1816	894	1.0∶1.9∶0.9	0.4

Pairs of adjacent COs were analyzed in two wildtype tetrads genotyped by sequencing (*wt*x29 and *wt*x30) and in 46 wildtype tetrads genotyped by microarray (Mancera, 2008). A list of all possible COs was first generated using CrossOver, with the threshold for merging closely spaced events set to 0 kb. This list of COs was then analyzed to determine how many chromatids were involved in each pair of adjacent COs. The expected ratio for true COs in wild-cells is 1∶2∶1, which reflects a lack of chromatid interference. Note that the total number of COs in the category "COs at least 5 kb apart" is slightly larger than the number of COs found when CrossOver is run with a 5 kb threshold for merging closely spaced events, due to merging of some COs in the latter case; the exact positions of merged COs are also different. For each pool of events, a chi-square test was performed to determine whether the observed ratio deviates significantly from the expected ratio of 1∶2∶1.

It is important to note that the categories of COs and GCs used for classification by CrossOver do not necessarily correspond to mechanistically distinct groups of events. These categories were created based on patterns of segregation that can be distinguished computationally, not on assumptions about their mechanistic underpinnings. Different events falling into the same category may have been created by different underlying processes; conversely, events in different categories may have arisen from similar processes. The categories are intended to serve as a framework for detecting a wide variety of possible changes in meiotic mutants.

CrossOver creates raw data files listing all individual events. It also produces a summary file listing key statistics, including the total CO and NCO count, average and median tract length for each type of GC, number of COs per chromosome, number of chromosomes lacking a CO, and several other parameters (see Materials and Methods). If multiple Seg files have been processed together, the output will include both per-tetrad and overall statistics. CrossOver also contains built-in functions to analyze multiple aspects of CO regulation. Distances between adjacent events are produced by the program, including inter-CO distances; based on these distances, CrossOver calculates the gamma and beta parameters used to estimate the strength of CO interference. A file containing the distances from centromeres or telomeres to COs and/or NCOs is also automatically produced, which can be used to assess repression of recombination in those regions. CrossOver also calculates the ratio of adjacent COs involving two, three, or four different chromatids, which provides a test of chromatid interference. Finally, the correlation coefficient between the total number of COs and NCOs per tetrad is calculated, which can be used to measure CO homeostasis.

### Sequencing of Two Wild-type Tetrads

We sequenced two wild type tetrads, *wt*x29 and *wt*x30, using the Illumina/Solexa Genome Analyzer II. For one tetrad, *wt*x29, multiplexing was used to reduce the cost of sequencing by running all four samples in a single lane. Each sequence read in this tetrad begins with a three-base “barcode” that arises from the adapter oligos used to construct the genomic DNA libraries. ReadAligner contains a function to sort the reads by barcode and remove the barcode before read alignment.

Read lengths for the two tetrads were also different (36 and 43 bp for *wt*x29 and *wt*x30, respectively) because the two samples were sequenced in different sequencing runs. We used ReadAligner and GenotypeCaller to analyze raw sequence reads from these tetrads. Table 2 shows the number of individual sequence reads that aligned to the reference genomes in each sample. The number of reads per spore and overall genome coverage were significantly lower in *wt*x29 than in *wt*x30 (∼6 fold vs. ∼26-fold average coverage) due to multiplexing and shorter read length. Therefore, a lower quality score threshold was used to make final genotype calls in this tetrad (see Materials and Methods). At this lower threshold, our simulations suggest that that fewer than 0.03% of markers are miscalled in a single spore (Table S6). Miscalling of even a small percentage of markers is a significant concern, as it has the potential to give rise to spurious single-marker NCOs. If 0.03% of 67, 583 markers are miscalled in each of four spores, this corresponds to about 80 wrong calls per tetrad. Of these, we would expect many to be excluded from the Seg file simply because any given position is only included in the Seg file if genotypes are assigned to all four spores at that position. For *wt*x29, 65% of markers received calls in all four spores; this number varies depending on the coverage of a particular experiment. Taking this into account, our simulation data still suggest about 50 wrong final calls per tetrad, of which the majority would be expected to appear as spurious single-marker NCOs. Each of our wild-type tetrads only had ∼30 single-marker NCOs. To determine whether a significant fraction of these resulted from wrong calls, we verified ten of them (five from each tetrad) by conventional Sanger sequencing and found that all ten had been correctly genotyped by ReCombine. Therefore, it appears that our simulations, which model genotype calling for a single spore, overestimate the true error rate in a full tetrad. We speculate that markers receiving a wrong genotype call may tend to be located in regions of the genome that are difficult to genotype, such as repetitive regions. The existence of hard-to-genotype regions is supported by the fact that in a given tetrad, a marker not assigned a genotype in one spore has a greater-than-average likelihood of not being assigned a genotype in the other three spores. In such regions, if an erroneous call is made in one spore at a given position, it would be highly unlikely that all four spores would receive a final genotype call at that position; thus, any wrong calls in these regions would tend to be excluded from the Seg file due to the requirement for a final genotype call at all four spores.

**Table 2:** Results of sequencing two wildtype tetrads.

Sample	# samples per lane	read length (bp)	# reads mapped to S288c or YJM789 (x10^6^)	Avg. fold genome coverage	% genome covered with >3 reads	#markers genotyped(x 10^4^)	Mean SNP coverage (SD)
*wt*x30a	1	43	7.1	25x	93	6.0	26 (7)
*wt*x30b	1	43	7.3	26x	93	5.8	26 (7)
*wt*x30c	1	43	7.3	26x	93	5.8	26 (7)
*wt*x30d	1	43	7.5	27x	93	6.0	26 (7)
*wt*x29a	4	33	3.0	8x	91	5.6	9 (3)
*wt*x29b	4	33	2.3	6x	86	5.3	6 (2)
*wt*x29c	4	33	2.4	6x	88	5.5	7 (3)
*wt*x29d	4	33	2.1	6x	83	5.2	6 (2)

Two wildtype tetrads *(wt*x30 and *wt*x29) were sequenced. The four spores within each tetrad are designated a, b, c, and d. Read lengths were different for the two tetrads because the samples were sequenced in different runs. For *wt*x29, barcoding was used to run all four samples in a single lane; raw read length was 36 bp before removal of the 3-base barcode. For *wt*x30, 55,988 markers were genotyped in all four spores. For *wt*x29, 41,782 markers were genotyped in all four spores.

**Figure 6:**
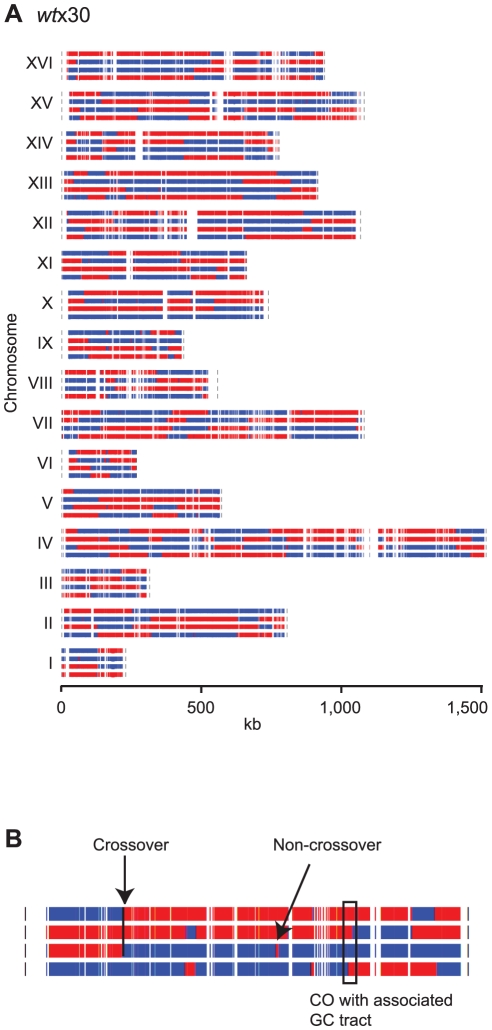
Segregation profile of a single wild-type tetrad. (**A**) Segregation of all 16 chromosomes in *wt*x30. Each group of four rows represents a single yeast chromosome, indicated by a Roman numeral on the left. Each marker genotyped in all four spores is indicated by a blue or red line (blue  =  S288c, red  =  YJM789). (**B**) Close-up view of chromosome 9 in this tetrad, showing several different types of recombination products.

Based on the markers genotyped in all four spores of each tetrad, we used GenotypeCaller to reconstruct the segregation profiles of both tetrads. Figure 6a shows the segregation pattern of *wt*x30 that was produced using plotTetradSeg, a component of the ReCombine package. A higher resolution view of chromosome 9 in this tetrad (Figure 6b) shows examples of a few different types of recombination products: a CO, a CO with an associated GC, and an NCO.

Next, we used CrossOver to identify and classify recombination events in the two sequenced wild-type tetrads. Table 3 lists several key results from this analysis. COs with simple associated GC tracts comprised, on average, 65% of all COs, and these GC tracts had an average tract length of 2.3 kb (±1.5 kb). As mentioned above, the interpretation of COs and GCs that occur very closely together, but are not directly connected, is not straightforward. For example, a GC tract occurring near a CO but not connected to it could be interpreted as either part of the same event (a Type 7 GC) or as an independent NCO (a Type 0 GC). Similarly, in some cases two COs that occur closely together could be interpreted alternatively as a single CO with an associated GC on a different chromatid (a Type 8 CO). CrossOver decides between these two possibilities by applying a user-defined range, set to 5 kb by default. Genotype changes occurring within 5 kb of each other are considered products of a single recombination event. Table 3 shows the results for both wild-type tetrads when the ranges for both closely spaced COs and closely spaced GCs is set to 0 kb or 2.5 kb rather than 5 kb. When these ranges are set to 0 kb, all GCs are considered independent events unless they are directly connected to a CO, and all reciprocal genotype changes are considered independent COs. Therefore, the reported number of COs per tetrad is higher when a 0 kb cutoff is used rather than 5 kb. As shown in Table 3, applying a 0 kb cutoff also increases the number of independent NCOs detected, and the average tract length of both NCOs and GCs is also impacted.

**Table 3:** Selected results of CrossOver analysis.

				Type I GCs: CO-associated, continuous			Type 0 GCs: NCO on one chromatid		
Strains	# of tetrads	range for merging close events (kb)	#COs per tetrad (SD)	# per tetrad (SD)	Avg. tract length (kb) (SD)	Median tract length (kb)	# per tetrad (SD)	Avg. tract length (kb) (SD)	Median tract length (kb)
*wt*x29	1	5	97	63	2.5 (1.5)	2.0	55	2.0 (1.7)	1.6
*wt*x30	1	5	99	64	2.2 (1.4)	1.9	44	2.2 (1.9)	1.7
*wt*x29+30	2	0	106 (2)	70 (2)	2.3 (1.4)	2.0	60 (7)	2.0 (1.7)	1.6
*wt*x29+30	2	2.5	104 (1)	69 (2)	2.3 (1.4)	2.0	56 (9)	2.2 (1.7)	1.7
*wt*x29+30	2	5	98 (1)	64 (1)	2.3 (1.5)	2.0	50 (6)	2.2 (1.8)	1.6
wt arrays	46	0	96 (9)	60 (8)	2.2 (1.9)	1.8	48 (9)	1.9 (1.6)	1.5
wt arrays	46	2.5	93 (9)	58 (8)	2.2 (1.9)	1.8	42 (8)	2.0 (1.6)	1.7
wt arrays	46	5	91 (9)	57 (8)	2.3 (1.9)	1.8	38 (8)	2.0 (1.7)	1.7

Analysis of *wt*x29 and *wt*x30 was performed either separately or together. “wt arrays” indicates the re-analysis by CrossOver of raw data from 46 tetrads genotyped by high-density tiling microarray (Mancera, 2008). "Range for merging close events" refers to the distance range used to determine whether an apparent double CO is actually a single event, and to determine whether a GC is considered "associated with" a nearby CO. A 5 kb range is used by default. Where applicable, the standard deviation for the measurement is given in parentheses.

### Genotyping a RM11-1a x S288c tetrad sequenced with 454 technology

To demonstrate the ability of ReCombine to handle data from other hybrid strains and other sequencing platforms, we obtained published data from a single RM11-1a x S288c tetrad sequenced on Roche-454 GS20 and GS20/FLX instruments [6]. Read lengths are generally longer on the 454 platform than on Illumina instruments; the average read lengths for the four spores in this experiment ranged from 107–191. The Bowtie short read aligner, which handles the core alignment function in ReadAligner, often fails to find alignments for long reads. This occurs because Bowtie does not allow gaps in alignments, which are more likely to occur in longer reads. As a result, the program performs best with shorter reads (approximately 50 bp or less) [10]. Other programs, such as BWA-SW [24] and SSHAHA2 [25], are better suited for alignment of long reads. Using ReadAligner, we found that only 32% of reads from the four spores in this experiment could be aligned to the merged RM11/S288c reference genome (compared to approximately 85% of reads in our Solexa/Illumina experiments with YJM789). To improve the number of reads aligned, we split the raw data into shorter reads of 50 bases or less. This improved the number of reads with valid Bowtie alignments to 48%. The average number of reads per spore in the raw data set was significantly smaller than in our experiments: approximately 350,000 reads per spore before splitting and 1.4 million after splitting. As result of the low number of reads aligned, the mean coverage level for each spore was only 1.8-3.4-fold in this experiment.

We created a list of expected polymorphisms between the RM11-1a and S288c strains based on their published genome sequences, and performed validation of the list using short sequence reads from the two parental strains sequenced by Qi et al [6]. Our list of polymorphisms included 42,106 SNPs, 2548 indels, and 3122 SNPs whose locations were known only in the S288c genome. We then used ReadAligner and GenotypeCaller to genotype all four spores at each of these positions. The resulting segregation profile is shown in Figure S2. Using CrossOver, we detected 91 COs, consistent with the results of Qi et al. However, due to the low coverage in this experiment, in order to detect all 91 COs we found it necessary to use a lower quality score threshold (50) than we would normally use. Even at this reduced threshold, only 8980 out of 47,776 markers could be genotyped in all four spores. In contrast, Qi et al. used a combination of BLASTN, CLUSTALW, and manual examination to align these reads and assign genotypes. They obtained 3.6-4.9-fold coverage per spore, compared to the 1.8-3.4-fold coverage we obtained with ReadAligner. The exact number of markers genotyped by Qi et al. in all four spores was not reported, but was most likely considerably higher than the 8980 markers we genotyped. Thus, although ReadAligner can handle reads from a variety of platforms (as long as the data are in fastq format), it is not the best tool for alignment of long reads. We were able to improve coverage by splitting reads into smaller units, but this is not an ideal solution, since it effectively reduces the amount of information available to assign alignment positions. It is also important to note that our analysis of the probability of errors in genotype calling (see Materials and Methods, Quality Score Threshold Selection) was based on the base-calling error rates of our Illumina/Solexa runs, and may not accurately predict the reliability of genotype calling when sequencing is performed on different platforms.

### Analysis of a Large Published Microarray Data Set by CrossOver

To demonstrate the utility of CrossOver in analyzing segregation data from sources besides sequencing, we obtained data from a previously published study in which 46 wild-type tetrads were genotyped by high-density tiling microarrays [5]. In these experiments, ∼52,000 markers were genotyped, with a median distance between markers of 78 bp, which is similar to the resolution of our sequencing data. Selected results are shown in Table 3 (“wt array tetrads”), again using three different distance ranges to demarcate closely spaced events. In the previously published analysis of these tetrads, the authors stated that they merged events occurring within 2.5 kb of one another. They reported an average number of 90.5 COs per tetrad. Our analysis of the same data set by CrossOver, using a cutoff of 2.5 kb, detected 93.1 COs per tetrad. The additional COs detected by CrossOver were distributed among 41 of the 46 tetrads, with each of those 41 tetrads having 1–7 extra COs in our analysis. Close examination of several of these extra COs revealed that most of them were closely spaced events located more than 2.5 kb but less than 5 kb apart, which were annotated as multiple COs by our method but merged into single events by Mancera et al, in spite of their stated intention to merge only events within 2.5 kb of each other. We detected an average of 47.8 NCOs per tetrad, a number that includes our Type 0, Type 5, and Type 6 GC tracts. Mancera et al. reported an average of 46.2 NCOs per tetrad. This number is lower partly because Mancera et al. merged closely spaced NCOs, whereas we do not. For comparison, we recalculated our results based on merging NCOs within 2.5 kb of each other, resulting in a slightly lower average of 47.1 NCOs per tetrad. The remaining difference in NCO counts between our analysis and theirs results mainly from differences in annotation of closely spaced events. Close examination of individual events revealed that Mancera et al. often annotated GCs near a CO as part of that CO event, even when the GC tract fell more than 2.5 kb away from the CO according to our calculations. We are unable to determine the exact underlying reasons for this discrepancy, but it may stem from differences in the way the positions of COs and/or GC tracts are calculated. We also found that, inexplicably, some (but not all) NCOs that spanned a single marker did not appear in their list of GC tracts.

Table 3 shows only a small subset of the results produced by CrossOver. The program produces summary statistics for each CO type and GC type shown in Figure 3, as well as lists of all individual events. The sorting of events into various types is considerably more sophisticated than previous work [4], [5], [6] in which GC tracts were classified simply as CO-associated GCs, NCOs, or complex GCs. This new classification system provides a framework for discovering specific changes in recombination outcomes in meiotic mutants.

**Figure 7:**
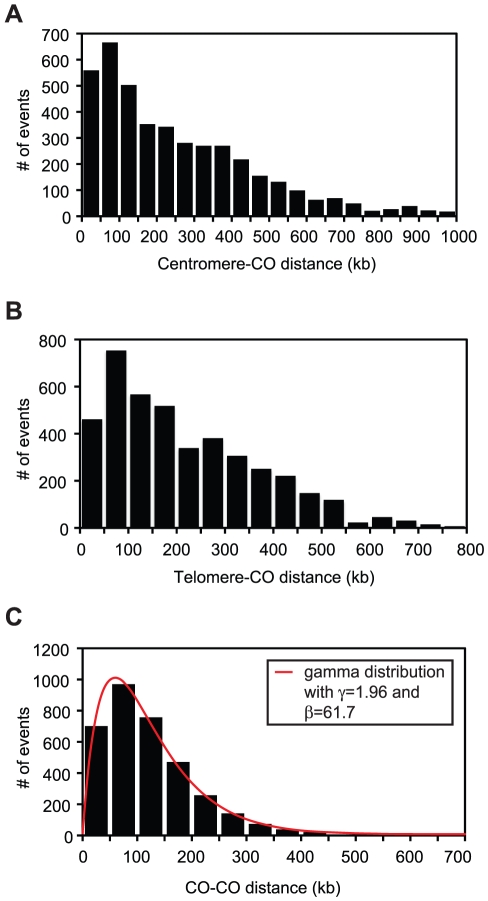
Analysis by CrossOver of 46 wild-type tetrads genotyped by microarray. Raw data were obtained from Mancera et al. [5]. All histograms show results obtained using a 5 kb range for closely spaced events. (**A**) Centromere-CO distances. The distance from each CO to the centromere was calculated by CrossOver and plotted as a histogram. (**B**) Telomere-CO distances. The distance from each CO to its nearest telomere was calculated by CrossOver and plotted as a histogram. (**C**) Inter-CO distances. A file containing distances between all pairs of adjacent COs was produced by CrossOver and plotted as a histogram. CrossOver also uses the list of inter-CO distances to calculate estimates of γ and β, the shape and scale parameters, respectively, for the gamma distribution. The red line shows a plot of the gamma probability distribution for γ = 1.96 and β = 61.7.

In addition to identifying and classifying individual recombination events, CrossOver also reports on several different aspects of the global distribution of recombination products. One salient feature of CO distribution in budding yeast is the repression of COs near centromeres and telomeres. To facilitate analysis of this aspect of CO regulation, CrossOver produces separate lists of the distances from each CO to its nearest chromosome end, and from each CO to the centromere. Figures 7A and 7B show plots of these distances for the 46 wild-type tetrads.

Another important influence on CO distribution is the phenomenon of CO interference, which refers to the observation that COs seldom appear closely together. The distances between pairs of adjacent COs can be modeled using the gamma probability distribution [16], [17].This distribution is characterized by a shape parameter, γ and scale parameter, β. Importantly, γ can be used as a measure of the strength of interference: a value of γ = 1 corresponds to no interference, while γ >1 indicates positive interference, and larger values of γ indicate stronger interference. CrossOver uses the distances between all pairs of adjacent COs to calculate estimates of γ and β. For the 46 wild-type tetrads analyzed here, the values calculated were γ = 1.96 and β = 61.7 when a 5 kb range was used to merge closely spaced events. When a 2.5 kb or 0 kb range was used to merge COs, the number of closely spaced COs increased and the apparent value of γ declined to 1.87 or 1.78, respectively. The value of 1.96 agrees well with previous estimates of γ in *Saccharomyces cerevisiae* [4], [26]. A plot of the gamma probability distribution function with these parameters is shown (Figure 7C) superimposed on the actual inter-CO distances. Note that unlike the plot of inter-CO distances in Figure 5, Figure 7C shows the results of CrossOver when a 5 kb range is used to merge closely spaced COs; therefore, it only includes distances between “true” COs, and not between products that could be derived from a single recombination event.

## Discussion

Whole-genome approaches have the potential to revolutionize the study of meiosis by allowing detailed analysis of recombination products on a genome-wide, cell-by-cell basis. However, the large amount of data generated by each experiment requires computational processing, which may pose a problem for geneticists lacking programming expertise. Here we have described methods that allow even researchers without deep knowledge of bioinformatics to perform genome-wide analysis of meiotic recombination in yeast.

Most previously published SNP and indel detection programs perform de novo SNP/indel calling, rather than deciding which of two expected alleles is present at a known set of marker positions. In de novo SNP calling, reads are aligned to a single reference genome, and any mismatches are considered possible SNPs. This is desirable when attempting to discover new polymorphisms or when all possible polymorphic loci are not known, for example when interrogating the human genome. Genotyping is typically performed by comparing the number (and in some methods, the quality scores) of “reference” vs. “variant” bases in reads aligning to a given position. In this procedure, the detection of variant alleles is limited by the fact that reads containing variant sequences often fail to align to the reference genome. Our procedure enhances the detection of variant positions (in our case, YJM789 alleles) by aligning reads to a merged reference genome containing both S288c and YJM789 sequences, thus capturing information from reads that would otherwise be discarded. The single previously published study of yeast meiosis by next-generation sequencing used a similar but distinct approach, performing a separate alignment to each reference genome [6]. However, the method of reconciling information from these two alignments to arrive at final genotype calls was not reported, so we are unable to perform a side-by-side comparison of the two procedures.

An additional confounding factor in de novo SNP detection methods is that some of the reads categorized as “variant” may not represent true polymorphisms, instead resulting from sequencing errors or misalignment of reads in repetitive or homologous regions. In our experiments, we have the benefit of knowing in advance the exact sequence and location of all possible SNPs and indels. We are thus able to reduce the confounding effect of spurious variant reads by specifically determining which of two expected bases is found at a given SNP position. Incorporating sequence quality scores into the genotyping method allows the user to control the stringency of genotype calls. Users can adjust the sensitivity and specificity of the method as needed for their own experiments.

The final component of our analysis package, CrossOver, extracts meaningful biological information from genotype data. CrossOver can process segregation profiles from various sources, regardless of the marker resolution. Detecting recombination events and sorting them into specific categories provides a basis for determining the effects of mutations on meiosis. By measuring changes in the prevalence or distribution of specific event types in meiotic mutants, we can begin to elucidate gene function. In addition, as new categories of events are discovered, the program can be altered to detect these new types. CrossOver provides an entry point into discovering patterns in the products produced by different recombination pathways, which can serve as signatures for those pathways in future experiments and provide insight into the molecular mechanisms underlying each pathway.

A feature of recombination that cannot currently be automatically analyzed by CrossOver is post-meiotic segregation (PMS). PMS refers to situations where recombination produces a region of heteroduplex DNA, with mismatches between the two strands at SNP or indel positions. PMS is detected by separating the two cells resulting from the first mitotic division after sporulation, and determining the genotypes of the resulting eight cells (four mother-daughter pairs). PMS events are identified by finding genotype differences between the mother and daughter cells in a pair. A recent study analyzed PMS on a genome-wide scale and found that approximately 9% of all recombination events in a wild-type tetrad showed PMS [27]. CrossOver does not currently have a designated function to associate PMS with the classes of recombinational repair. However, the program FindPMS, included in the ReCombine package, can be used to detect PMS events in four mother-daughter pairs that are genotyped as two separate tetrads. Users can sort the PMS events into categories by comparing their locations to the positions of recombination products found in the individual tetrads by CrossOver. Since the number of PMS events is low (6–18 per tetrad), manual curation of these events is feasible.

## Materials and Methods

### Generation of sequencing data

Genomic DNA libraries were prepared as described in the Illumina protocol “Preparing Samples for Sequencing Genomic DNA.” Samples were sequenced at the Vincent J. Coates Genomics Sequencing Laboratory of the California Institute for Quantitative Biosciences at the University of California, Berkeley, or at the Center for Advanced Technology at the University of California, San Francisco. To reduce the cost of sequencing, for tetrad *wt*x29, multiplex sequencing was used to sequence multiple yeast samples in one lane. Short fragments of yeast genomic DNA were ligated to adapter oligos that contained one of four three-nucleotide “barcodes”: TGT, CAT, ACT, and GTT [28]. Libraries with different barcodes were pooled together in equimolar ratios for sequencing.

### Experimental Design

As high-throughput sequencing technology evolves, it is becoming possible to multiplex greater numbers of samples and still obtain sufficient coverage to map meiotic recombination. An approximation of the expected coverage from a given experiment can be calculated by multiplying the expected number of reads per lane by the read length and by the expected percentage of usable reads. (In our experiments, approximately 85% of reads could be aligned to the reference genome; this may vary depending on the sequencing platform and sample preparation protocol used.) The resulting number of bases covered should then be divided by the size of the yeast genome (12 Mb) to yield the fold coverage. For example, for a sequencing platform capable of generating 10 million 50-base reads per lane, the expected coverage per lane would be [(10×10^6^ reads)×(50 bases per read)×(0.85 reads aligned)]/(12×10^6^ bases in yeast genome)  = 35-fold coverage. We have successfully genotyped tetrads with as low as 6-fold average coverage; however, as coverage declines, the ability to detect recombination events also declines. Refer to Table S6 for estimates of the sensitivity and specificity of genotype calling at different coverage levels.

The number of tetrads required to detect changes in recombination will vary depending on the magnitude of the change. For example, based on bootstrapping analysis, we previously determined that ∼250 intercrossover distances is sufficient to distinguish between a strain with wild-type crossover interference and a strain that has completely lost interference [4]. However, 250 intercrossover distances is not sufficient to detect a moderate loss of interference. Standard statistical tests, such as chi-square or t-tests, should be applied to determine whether changes are significant.

ReadAligner can accept data from other platforms besides Illumina/Solexa, as long as the input file is in fastq format. Read quality scores in the fastq file may be in Sanger or Illlumina format; ReadAligner allows the user to select the quality score format. The Bowtie program, which performs the alignment of raw reads in ReadAligner, can handle read lengths of up to 1024 bases. However, since Bowtie alignments are not tolerant of indels, which become more prevalent with increasing read length, Bowtie is best suited for short reads (roughly 50 bases or less; described in detail in [10]). At longer read lengths, the percentage of reads that can be aligned to the reference genome decreases.

ReadAligner does not currently support paired-end alignment. Data from paired-end sequencing experiments should be run as separate (unpaired) reads. Advanced users may modify the Bowtie input parameters in ReadAligner to support paired-end alignment.

### Software implementation

All programs except for those used for segregation plots were written in Python and are compatible with Python version 2.6 (or later versions in the 2.x series). The plotting programs were written in R and are compatible with R Version 2.11.0 or higher. The numerical package NumPy 1.3.0 (http://numpy.scipy.org) was used for a variety of statistical analyses. ReadAligner relies on the Bowtie short read aligner [10]. The ReCombine user manual and software package are available for download at http://sourceforge.net/projects/recombine/.

### Reference genome sequences

The S288c genome sequence is complete and fully assembled [29] (Genbank accession numbers NC_001133 through NC_001148). The published genome sequence of YJM789 (Genbank accession number AAFW02000000) is not fully assembled; it is organized into large contigs rather than complete chromosomes [19], [20]. The RM11-1a genome was obtained as supercontigs 1.1–1.17 from the Broad Institute, Cambridge, MA. Files containing the individual and merged reference genomes used in this study are included in the downloadable ReCombine package.

### SNP and indel list creation

A critical first step in genotyping SNPs and indels is the creation of a “SNP list” and “indel list” containing all expected sequence differences between the two parent yeast strains. By aligning the YJM789 sequence contigs against the fully assembled S288c genome, Wei et al. reported ∼60,000 single-nucleotide polymorphisms (SNPs) and ∼6,000 insertions or deletions (indels) between the two strains [20]. We validated this list by sequencing the S96 and YJM789 haploid parents used in our lab (data not shown). We discovered the loss of polymorphism at 1,081 published SNP locations, where the two parental strains no longer have a sequence difference at the reported SNP position in our yeast strains. Therefore, these positions do not appear in our SNP list. We encountered difficulties in reconciling nearly half of the indels reported by Wei et al. For many of the reported indels, the sequence given in the published indel list does not match the published sequence of the S288c reference genome at that position. Out of the ∼6,000 indels reported by Wei et al, we were able to verify the position and sequence of 3,401 indels in the S288c reference genome.

Many telomere-proximal regions are missing from the published sequence of YJM789. We compiled a list of SNPs in these regions based on our sequencing of the YJM789 strain. Since the positions of these SNPs is not known in the YJM789 reference genome, two separate SNP lists were used in our analysis: one containing SNPs and indels with known positions in both reference genomes, and one containing telomeric SNPs, whose position is known only in the S288c reference genome (Table S7).

All SNP and indel markers were subjected to the following additional validation procedure to remove any markers that could not be used reliably to distinguish between the two genotypes. We used sequencing data from each individual haploid parent, and aligned it against the merged reference genome using ReadAligner to generate S288c and YJM789 count files (exactly as described for meiotic progeny). We then analyzed the cumulative quality score for each marker position as follows. If, for a given marker position, the cumulative score for reads from the wrong parent aligning to a given reference genome was greater than or equal to half the score for the correct parent, we considered the marker unreliable and eliminated it from the list. This resulted in the removal of 319 SNPs and 593 indels. Altogether, after elimination of unreliable markers, our marker lists contained a total of 67,583 polymorphic markers, including 64,161 SNPs, 2,785 indels, and 637 new SNPs found near chromosome ends. For genotyping the RM11-1a x S288c tetrad, a SNP/indel list was produced using Mummer 3.22 [30] and validated using sequencing data from each haploid parent. These lists are included in the ReCombine package available for download from SourceForge.

### Read alignment by ReadAligner

The input for ReadAligner is a fastq file containing short sequence reads and quality scores. If multiple samples have been sequenced in one lane, the reads must be sorted into separate pools based on the barcode at the beginning of each read and trimmed of the barcode bases before alignment. ReadAligner contains a function to accomplish this. ReadAligner then uses the Bowtie short read aligner [10] to align reads to a merged reference genome containing both the reference genomes. By default, up to two mismatches are tolerated within the first 28 bases of the read, and no gaps are allowed in Bowtie alignments. Reads with more than two valid alignments to the merged genome are discarded. This criterion ensures that only reads that can be unambiguously mapped to a specific location in the genome are used for further analysis. Bowtie alignment results for the merged reference genome are then separated into two pools corresponding to the parent genomes, S288c and YJM789, for further analysis. The number of S288c-aligning or YJM789-aligning reads covering each SNP or indel position is listed in “Count files,” (Tables S1 and S2). There are separate Count files for SNPs and indels. Note that it is possible, though not common, for a read to align to one genome but match the sequence of the other; this usually occurs because Bowtie does not guarantee that the reported alignment is the best possible one if all valid alignments contain mismatches, particularly if they occur in the right (low-quality) end of the read. Therefore, as well as tabulating the number of reads covering a given SNP or indel position, the count file also records whether the sequence of each read at each SNP or indel position matched the S288c or YJM789 reference genome.

A second Bowtie alignment is carried out in which the reads are aligned only to the S288c genome. This alignment is used to genotype telomeric SNPs, since these regions are not included in the published sequence of the YJM789 genome. In this step, any reads with more than one valid alignment are discarded. In total, five count files are produced from each sequencing sample: three S288c Count files (listing SNPs, indels, and telomeric SNPs, respectively), and two YJM789 Count files (listing SNPs and indels, respectively).

### Genotyping of SNPs

Information contained in the Count files is used to assign a provisional genotype call to each position; this is carried out separately for reads aligning to each reference genome. These two provisional calls are later reconciled by GenotypeCaller, yielding a single final call for each marker.

For each set of reads aligning to a particular SNP in a given reference genome, the bases that align to the SNP position are sorted into three possible base calls: S288c, YJM789, or other. (For example, if a SNP position is expected to have a C in the S288c genome and a T in the YJM789 genome, possible base calls would be “C,” “T,” and “other.”) A calculation is then performed using the quality scores assigned to the specific base (or bases) in each sequence read corresponding to the SNP position. The Solexa/Illumina Pipeline software computes a base quality score for each base of every Illumina sequence read using an error model generated from a control sequencing lane with a control template [21]. These quality scores are listed in the fastq input files.

We use these base quality scores to calculate a cumulative quality score for each of the possible genotypes at each SNP position, as follows. For each genotype, a “nucleotide score” is calculated as the difference between the sum of the base quality scores of the reads with that particular nucleotide and the sum of the base quality scores of all other reads that did not have that particular nucleotide. In the previous example, the S288c score would be the sum of the base quality scores for that C in reads aligning to that position, minus the sum of the base quality scores for all other nucleotides aligning to that position.

For each sequencing sample, a quality score threshold is set by the user (see below for details about choosing the quality score threshold). For each marker position, if the cumulative quality score of one of the possible genotypes passes the threshold, the position is provisionally assigned that genotype (these initial calls are referred to as “provisional” because they are assigned based on reads aligning to one reference genome at a time. The two provisional calls are then reconciled at the next stage of analysis). For indels, a similar procedure is carried out, but with modifications; details are given below.

The provisional genotypes determined as described above are recorded in Master files (Tables S3 and S4). Multiple sets of master files are produced, each using a different quality score threshold: 20, 50, 80, 100, 120, 150, 200, 250, 300, 400, and 500. In addition, as for count files, there are separate Master files for SNPs, indels, and telomeric SNPs.

### Genotyping of Indels

Marker quality scores for indels are calculated in a similar manner to the SNP markers with the following modifications. Since an insertion may span several bases, one reference base is selected for the purpose of calculating the quality score for that insertion. The reference base for an insertion is the center base of an odd-length insertion or the right-most base of the two center bases of an even-length insertion. For a deletion, the marker quality score is calculated using the average of the base quality scores of the two bases directly bordering the deleted region. To ensure that the two bases directly bordering the deleted region are adjacent bases on a sequence read and that no insertion is present between them, the first and last three bases of all sequence reads are not included in this analysis. This is based on the fact that since Bowtie tolerates up to two mismatches in the high-quality end of a read, it is possible for a read with no sequencing errors to match at an indel site if two or fewer bases lie on one side of the indel.

### Quality Score Threshold Selection

We performed simulations to test the sensitivity and specificity of genotype calling using various quality score thresholds. A section of ∼700 kb of chromosome 2 was used for the simulations. A chimeric chromosome consisting of sequences from both reference genomes (S288c and YJM789) was generated by introducing a crossover every 140 kb (five COs total) which is the number found experimentally for that chromosome. The open source Maq software [12] was used to randomly generate a pool of simulated reads, introducing errors in the reads based on position-specific error probabilities taken from an actual Solexa/Illumina run. Due to the variety of genome coverage levels and read lengths in our real experiments, the simulation was performed for a number of conditions. Table S6 lists the various coverage levels and read lengths simulated. The simulation was repeated 6 times at each coverage level.

At each coverage level, simulated reads were aligned to the merged reference genome using ReadAligner and Master files were produced for the following marker threshold levels: 20, 50, 80, 100, 120, 150, 200, 250, 300, 400, and 500. Only SNPs were used in this analysis; indels were not included. The final marker genotype calls at every threshold were compared to the actual marker genotypes from the chimeric chromosome used in the simulation. The number of incorrectly genotyped markers in each simulation at each coverage level and threshold was calculated. Note that this procedure mimics genotyping of a single spore, not an entire tetrad. Table S6 shows the percentage of correctly and incorrectly genotyped markers. In practice, we normally choose the threshold that gives the highest percentage of correctly identified markers with the following constraints: a) the average number of incorrectly indentified markers in the chimeric chromosome must be less than or equal to 3, and b) threshold levels 20 and 50 are not considered due to the low number of reads (1 or 2 reads) required to reach the thresholds.

### Final genotype determination by GenotypeCaller program

GenotypeCaller uses Master files as input. The user determines the quality score threshold to be used and chooses the corresponding set of Master files to use as input. Thresholds used for the two wild type tetrads in this study were 80 for *wt*x29 and 150 for *wt*x30. GenotypeCaller then determines final marker genotype calls by comparing the provisional calls from the two reference genomes listed in the Master files. The rules used to reconcile differing calls are as follows. For SNPs, positions with agreeing genotype calls from reads aligning to both reference genomes are given that genotype. SNPs with conflicting genotype calls from the two reference genomes are discarded. For SNPs in which only one reference genome yields a genotype, a final SNP genotype is determined only if the genotype call matches the reference genome from which it originated. For example, if a marker is genotyped as S96 according to the Bowtie alignment to the S288c reference genome and not genotyped in the alignment to the YJM789 reference genome, then the SNP is given a final marker call of the S96 genotype. This criterion follows from the fact that reads containing S96 SNPs should preferentially match to the S288c reference genome, rather than the YJM789 reference genome. The reverse is true for reads containing YJM789 SNPs. Indel genotyping follows a slightly different procedure. Since a deletion in one reference genome is an insertion in the opposite reference genome, an ideal indel displays a strong preferential alignment between the two genomes, resulting in a high quality score in one genome and a low quality score in the opposite genome. Therefore, when making genotype calls for indels, we require that in addition to passing the quality score threshold, the cumulative quality score of the reads aligning to one genome must be at least twice the cumulative quality score of reads aligning to the same indel position in the other genome.

After determining final genotype calls, GenotypeCaller compiles a list of markers genotyped in all four spores of a tetrad. A Seg file is produced containing only these markers, along with the genotypes of all four spores at each of these positions (Table S5).

### Plotting segregation profiles

The ReCombine package includes two R scripts, plotTetradSeg and plotTractSeg, which can be used to create graphical representations of tetrad segregation. plotTetradSeg displays the segregation of an entire tetrad, while plotTractSeg can be used to display a region defined by any two desired markers. Both programs use the Seg file as input.

### CrossOver program

#### Input file format

As input, CrossOver accepts seg files produced by GenotypeCaller, or by the program Allelescan [7]. Segregation data from other sources can also be used, as long as the data are properly formatted. The detailed format of each line of data should be as follows:

5\t391664\t\t0\t1\t1\t0\r\n (Allelescan output format)

or

5\t391664\tspaceholder\t0\t1\t1\t0\n (GenotypeCaller output format).

(The above example describes a SNP on chromosome 5, position 391664, in which the genotypes of the four spores are 0, 1, 1, and 0, respectively.).

#### **Identification of recombination events**

A detailed flow chart showing the steps carried out by CrossOver is shown in Figure S3. Initially, the program sets aside any markers with non-2∶2 segregation ratios and considers only markers segregating 2∶2. COs are identified as locations where markers undergo a reciprocal genotype switch (Figure S3). In most cases, only two of the four chromatids undergo a switch; however, if four chromatids undergo a switch at the same location, this is classified as a double CO and is considered separately (CO Types 5, 6, and 7). The program then determines whether there are intervening non-2∶2 markers between the markers flanking the CO. If so, these are classified as GC tracts associated with a CO. If not, the CO is classified as a CO without detectable GC tract (CO Type 0). COs are placed into different categories depending on whether the associated GC tract lies on one of the chromatids involved in the crossover, on a non-crossing-over chromatid, or both (CO Types 1, 2, and 3). The GC tract itself is categorized as GC Type 1 or 6. If a CO with an associated GC does not fit easily into one of the preceding categories, it is placed into CO Type 4 (or Type 7, if it is a double CO). These are typically COs with discontinuous GC tracts.

After COs have been identified, CrossOver identifies pairs of COs that occur within a user-defined range (set to 5 kb by default). Only single COs are included in this analysis. When three or more COs occur within 5 kb of one another, a message appears on the console instructing the user to manually inspect the region.

If two closely spaced COs involve the same two chromatids, the two COs are reclassified as a single NCO (GC Type 5). If three chromatids are involved, the two COs are reclassified as a single CO with an associated GC on a non-crossing-over strand (CO Type 8). Note that CO Types 3 and 8 are similar in that both contain a GC tract on a non-crossing-over chromatid. The key difference is that Type 3 COs contain a 4∶0 tract, whereas Type 8 COs do not. CO type 2 is also similar to CO Types 3 and 8. Although all three can be distinguished computationally, we do not know whether they arise from the same underlying biological process.

After all COs have been identified and sorted, non-2∶2 markers are separated into two lists: those that segregated 3∶1 or 1∶3, and those that segregated 4∶0 or 0∶4 (Figure S3). GC tracts are then identified in each list by grouping consecutive markers together. A single non-2∶2 marker without adjacent non-2∶2 markers is considered a tract of its own. Any GC tracts involving the first or last marker of a chromosome are categorized as GC Type 3 or 4. GC tracts falling within a user-defined range of a CO are then identified; by default, this range is set to 5 kb. GC tracts falling within this range are classified as GC Type 6 or 7 depending on whether or not the tract occurs on a chromatid involved in the CO. The CO near a Type 6 or 7 GC may, in addition, have a GC directly connected to it (on the chromatids involved in the CO). If such a tract is present, it is classified as GC Type 1 and also as Type 8 (if it occurs in conjunction with a Type 6 GC) or Type 9 (if it occurs in conjunction with a Type 7 GC). GC tracts with 3∶1 or 1∶3 segregation that do not occur near a CO are classified as NCOs (GC Type 0). GC tracts with 4∶0 or 0∶4 segregation that do not occur near a CO are classified as 4∶0 tracts (GC Type 2). Note that 4∶0 tracts that occur as part of a Type 3 CO do not appear in the GC Type 2 category.

#### **Calculation of CO and GC positions**

For a simple CO without an associated GC, the CO position is calculated as the midpoint of the two markers defining the genotype switch (Figure S4). In cases where a CO has an associated GC on a crossing-over chromatid, the program first finds the midpoint of the markers defining the genotype switch on each chromatid, then calculates the CO position as the midpoint of these two midpoints. Detailed examples of how CrossOver determines the positions of COs and GCs are shown in Figure S4. In general, the length of a GC tract is calculated by finding the difference between these two midpoints. A minimum and maximum possible tract length is also calculated by using the innermost or outermost markers, rather than midpoints. For GC Type 5, which consists of two GC tracts on different chromatids, a single tract length is reported; this is the distance between the midpoint of the two markers defining the 5’ end of the leftmost tract and the midpoint of the two markers defining the 3’ end of the rightmost tract. For GC Types 6 and 7, there are often two component GC tracts: one that is contiguous with the CO, and another that is not directly connected to the CO. In the GC tract report, GC Types 6 and 7 list only the tract that is not directly connected to the CO. The GC tract that is contiguous with the CO, if present, is reported as a Type 1 GC, and it is also reported as Type 8 or 9 (depending on whether it is part of a Type 6 or 7 GC event, respectively). Therefore, in the GC tract report, most Type 6 and 7 GCs have a nearby Type 1 GC, and that Type 1 GC is also listed as a Type 8 or Type 9 GC. An additional category of GCs not shown in Figure 3 is GC Type 10, which consists of only those Type 1 COs that are not also classified as Type 8 or 9.

For double COs (CO Types 5, 6, and 7), it is impossible to determine which chromatids were involved in each of the two exchanges. Therefore, CrossOver randomly pairs the possible partners.

#### Output

CrossOver creates a raw data file that contains a list of every CO found. This list identifies the chromosomal position, the CO type, the identities of the two chromatids involved in the exchange, and if applicable, the tract length of any associated GC and the number of markers defining the GC. A separate file contains all GC tract data. For each GC tract the position, GC type, estimated tract length, minimum and maximum possible tract lengths, number of markers involved, positions of the first and last markers within the GC tract, and the number of YJM789 alleles found in the first marker of the tract (for analysis of allele parity) is noted. CrossOver also creates summary reports detailing key statistics, which include but are not limited to: total CO number, number of COs per chromosome, number of each type of CO and GC, number of nonexchange chromosomes (E0s), and average and median GC tract lengths. If multiple tetrads have been analyzed in batch, these statistics include per-tetrad results. Distances between adjacent events are also produced by the program, including inter-CO distances, inter-NCO distances, and the distances from centromeres or telomeres to COs and/or NCOs. Results also include the gamma and beta parameters used to calculate the strength of CO interference; the ratio of adjacent COs involving two, three, or four different chromatids, used to evaluate chromatid interference; and the correlation coeffcient between the total number of COs and NCOs per tetrad, which can be used to measure CO homeostasis.

## Supporting Information

Figure S1 **Use of quality scores in genotype calling.** This example shows reads from spore C in tetrad *wt*x30 aligning to a particular SNP on chromosome I. In the top panel, reads whose reported “best” alignment falls within the S288c reference genome are shown. These reads are tabulated in the S288c count file for this spore. The next panel shows reads whose “best” alignment falls within the YJM789 genome. These reads are tabulated in the YJM789 SNP count file for this spore. The quality scores shown are the scores assigned to the nucleotide at the SNP position in each read by the Solexa/Illumina pipeline. For reads aligning to each reference genome, a cumulative score for each possible genotype is calculated. This is done by first adding the quality scores for all reads with a given genotype; this sum is listed in the corresponding count file. From this sum, the quality score sums for all other possible genotypes are subtracted, yielding the cumulative quality scores shown here. If one of these cumulative scores passes the quality score threshold chosen for the experiment, then the genotype is “provisionally” called as that genotype. This provisional call is listed in the master file. If more than one genotype passes the threshold, or if no genotypes pass the threshold, a provisional call of “neither” is made. Finally, the two provisional calls are reconciled to yield a single file genotype call, recorded in the Seg file.
(EPS)

Figure S2 **Segregation profile of an RM11-1a x S288c tetrad. (A)** Segregation of all 16 chromosomes in the RM11-1a x S288c tetrad sequenced by Qi and co-workers (Qi, 2009). Each group of four rows represents a single yeast chromosome, indicated by a Roman numeral on the left. Each marker genotyped in all four spores is indicated by a blue or red line (blue  =  S288c, red  =  RM11-1a).
(EPS)

Figure S3 **Detailed CrossOver Pipeline.** The figure shows the logic used to find recombination events and sort them into categories. Note that in many cases, a single event contains both a CO and a GC; these are indicated on the figure. In Steps 7–11, a segregation matrix is created in which [a, b, c, d] contains the number of genotype switches on each of the four chromatids in the interval defined by the two ends of the CO. For example, in Step 8, [0, 0, 1, 1] indicates that two chromatids have no genotype changes and two chromatids have a single genotype change (either from 0 to 1 or 1 to 0) in that interval.
(EPS)

Figure S4 **Examples of how CO and GC events are identified.** Part of a seg file is shown in each case. Genotype swtiches are indicated by gray boxes. **(A)** A CO without associated GC (Type 0 CO). The CO position is calculated as the midpoint between the two markers defining the genotype switch. **(B)** A CO with associated GC (Type 1 CO and Type 1 GC). A region of 3∶1 segregation appears between the two markers defining the ends of the CO. Each end of the GC is defined as the midpoint between the two markers with opposite genotypes. GC tract length is found by calculating the distance between these two midpoints, and the CO position is calculated as the midpoint between the two midpoints. A minimum and maximum GC tract length is also calculated by using the innermost or outermost marker pairs to define the ends of the GC tract. The number of markers (in this case, 2) falling within the GC tract is also recorded. **(C)** A CO with associated GC on a chromatid not involved in the CO (Type 2 CO and Type 6 GC). The CO occurs between spores 2 and 3 and the GC is in spore 1. **(D)** A GC involving a chromosome end. In this example, the 3∶1 tract is in spore 4, beginning at the third marker in this list and continuing through the last marker genotyped on this chromosome.
(EPS)

Table S1 **Examples of SNP count files.**
(XLS)

Table S2 **Examples of indel count files.**
(XLS)

Table S3 **Example of a SNP master file.**
(XLS)

Table S4 **Example of an indel master file.**
(XLS)

Table S5 **Example of a seg file.**
(XLS)

Table S6 **Quality score threshold simulation results.**
(XLS)

Table S7 **Examples of SNP and indel lists.**
(XLS)
